# Characterization of cell death inducing *Phytophthora capsici* CRN effectors suggests diverse activities in the host nucleus

**DOI:** 10.3389/fpls.2013.00387

**Published:** 2013-10-21

**Authors:** Remco Stam, Andrew J. M. Howden, Magdalena Delgado-Cerezo, Tiago M. M. M. Amaro, Graham B. Motion, Jasmine Pham, Edgar Huitema

**Affiliations:** ^1^Division of Plant Sciences, College of Life Sciences, University of Dundee, Dundee, UK; ^2^Cell and Molecular Sciences, The James Hutton Institute, Dundee, UK; ^3^Dundee Effector Consortium, The James Hutton Institute, Dundee, UK

**Keywords:** *Phytophthora capsici*, effector, CRN, nucleus, cell death, immunity

## Abstract

Plant-Microbe interactions are complex associations that feature recognition of Pathogen Associated Molecular Patterns by the plant immune system and dampening of subsequent responses by pathogen encoded secreted effectors. With large effector repertoires now identified in a range of sequenced microbial genomes, much attention centers on understanding their roles in immunity or disease. These studies not only allow identification of pathogen virulence factors and strategies, they also provide an important molecular toolset suited for studying immunity in plants. The *Phytophthora* intracellular effector repertoire encodes a large class of proteins that translocate into host cells and exclusively target the host nucleus. Recent functional studies have implicated the CRN protein family as an important class of diverse effectors that target distinct subnuclear compartments and modify host cell signaling. Here, we characterized three necrosis inducing CRNs and show that there are differences in the levels of cell death. We show that only expression of CRN20_624 has an additive effect on PAMP induced cell death but not AVR3a induced ETI. Given their distinctive phenotypes, we assessed localization of each CRN with a set of nuclear markers and found clear differences in CRN subnuclear distribution patterns. These assays also revealed that expression of CRN83_152 leads to a distinct change in nuclear chromatin organization, suggesting a distinct series of events that leads to cell death upon over-expression. Taken together, our results suggest diverse functions carried by CRN C-termini, which can be exploited to identify novel processes that take place in the host nucleus and are required for immunity or susceptibility.

## Introduction

Within the natural environment, plants are continuously challenged by a diverse array of microbes that can cause disease, including bacteria, fungi, and oomycetes. In order to counteract infection, plants have evolved the ability to recognize Microbe or Pathogen Associated Molecular Patterns (MAMPs or PAMPs, respectively) through Pattern Recognition Receptors (PRRs) localized in the host cell membrane. This recognition of PAMPs in turn activates PAMP-Triggered Immunity (PTI), preventing establishment of disease (Zipfel, 2008; Monaghan and Zipfel, 2012). In a select few cases, pathogens successfully infect plants by either limiting PAMP perception or perturbing PTI by interfering with signal transduction or associated cellular processes required for effective host immune responses (Jones and Dangl, 2006; Zipfel, 2008). This implies that pathogens have evolved molecular strategies to evade or circumvent host immunity. Consequently, host-pathogen interactions are considered dynamic associations featuring specialized pathogen machineries that aim to suppress (inducible) immune responses.

Key to understanding the mechanisms by which pathogens evade or suppress plant immune responses has been the identification of secreted proteins, termed effectors, which have been found in virtually all pathogen genomes studied to date (Hogenhout et al., 2009; Stergiopoulos and de Wit, 2009; Hann et al., 2010; Oliva et al., 2010). In some cases, effector activities toward virulence have been demonstrated and linked to host susceptibility, supporting the notion that effectors can trigger susceptibility on their hosts [Effector Triggered Susceptibility (ETS)] (Bos et al., 2010; Yeam et al., 2010). Consequently, models have now emerged which describe secreted effector proteins that upon delivery to host cellular compartments, modify their targets and trigger susceptibility (Howden and Huitema, 2012). Besides PRR mediated responses, plants have acquired another layer of immunity. Most plants carry another class of receptors (termed Nucleotide Binding-Leucine Rich Repeat proteins or NB-LRRs), which reside inside host cells and upon recognition of cytoplasmic effectors, trigger immunity (Effector Triggered Immunity, ETI). With an increasing number of PRRs, PAMPs, effectors and NB-LRRs identified and characterized, observations suggest that both secreted pathogen proteins together with host receptors and signaling protein repertoires, determine interaction outcomes at the early stages of infection.

In recent years, a body of evidence has emerged which implicates the nucleus as a key cellular compartment in which the fate of host-pathogen interactions is determined (Liu and Coaker, 2008; Deslandes and Rivas, 2011; Rivas, 2012). In agreement with this, host protein classes with diverse functions have been shown to function in the nucleus toward immunity. These include plant disease resistance proteins, mitogen-associated protein (MAP) kinase signaling components, and transcription factors that collectively operate to regulate defence response genes following pathogen perception (Kinkema et al., 2000; Pandey and Somssich, 2009; Deslandes and Rivas, 2011; Park and Ronald, 2012; Rasmussen et al., 2012). In some cases, the mechanisms of activation are known and a major emerging theme is the exchange of key regulators and cellular signals between the cytosol and host nucleus (Shen and Schulze-Lefert, 2007). These processes generally result in the activation of defence responses and initiation of transcriptional programmes that elevate resistance. Given the role of the nucleus in plant defences and the ability of pathogens to suppress immunity, the view has emerged that perturbation of nuclear signaling by means of secreted pathogen effectors, may form an important virulence strategy to achieve disease.

Plant pathogenic oomycetes form a distinct lineage of eukaryotes that cause devastating diseases on a wide range of plants important to agriculture, forestry and natural ecosystems. For example, *Phytophthora infestans*, the causal agent of late blight on potato and tomato continues to cause hardship throughout the world with multi-billion dollar losses each year (Lamour et al., 2007). Other economically devastating pathogens include *P. sojae* and *P. capsici*, the major disease-causing agents on soybean and pepper, respectively. The shear economic impact that this group of pathogens incites has been, and continues to be, a driving force in our quest to understand *Phytophthora* parasitism.

Plant pathogenic oomycetes harbor a diverse class of effectors, termed the Crinklers (CRNs). All CRN proteins feature a conserved N-terminal domain specifying translocation and diverse C-terminal regions carrying distinct effector functions (Schornack et al., 2010). Crucially, a considerable number of CRN proteins have been identified in the genomes of all plant pathogenic oomycetes examined to date (Tyler et al., 2006; Gaulin et al., 2008; Haas et al., 2009; Lévesque et al., 2010; Schornack et al., 2010; Links et al., 2011; Lamour et al., 2012; Stam et al., 2013), suggesting that they have important roles in oomycete pathogenesis on plants.

Localization studies on diverse sets of CRN effectors from divergent oomycete species revealed they all accumulate in the host nucleus upon ectopic expression in plants (Schornack et al., 2010; Stam et al., 2013). These observations combined with the identification of (cytoplasmic) RXLR effector proteins that target the nucleus (Dou et al., 2008; Caillaud et al., 2012; Qiao et al., 2013) suggest that plant nuclear processes must present an important target for filamentous pathogens to achieve virulence (Birch et al., 2006; Morgan and Kamoun, 2007; Schornack et al., 2009). If true, nuclear effectors would carry the activities that allow modification of nuclear signaling networks and suppression of plant defences, providing useful tools for understanding the role of the plant nucleus during immunity.

CRN proteins were initially identified through their ability to cause crinkling and necrosis upon expression in plant tissue, and consequently this protein family is generally considered as a class of cell death inducing effectors (Torto et al., 2003). Recent studies, however, show that this is not a universal feature of either CRN proteins or their C-terminal effector domains. Expression of CRN effector domains leads to cell death in only a select few cases, suggesting diverse activities underpinning effector function (Haas et al., 2009; Schornack et al., 2010; Stam et al., 2013). Importantly, despite inducing cell death upon ectopic expression, infection assays revealed that only one CRN effector promotes virulence. Localization studies revealed distinct subnuclear localization patterns, further suggesting diverse functions in plants leading to cell death (Stam et al., 2013). In this paper, we expand on our work on CRN effectors and provide evidence suggesting diverse molecular events leading to cell death in plants. Comparative analyses between three necrosis-inducing CRN effector domains (DN17, D2, and DXZ) revealed differences in the timing and occurrence of cell death in *N. benthamiana*. Consistent with diverse effector activities, we show that expression of only one CRN domain has an additive effect on PAMP-induced cell death, suggestive of distinct effector induced perturbations affecting different nuclear processes. Confocal and OMX 3D-SIM microscopy on living cells substantiated these observations by showing distinct subnuclear localization patterns for each cell death inducing effector and crucially, specific effector induced changes in nuclear morphology, possibly leading to cell death. Taken together, our results suggest diverse functions carried out by CRN C-termini in the host nucleus that lead to cell death. We conclude that although cell death induction may not be a direct virulence function, it may represent an important phenotypic outcome, suited to study effector and target functions. A firm understanding of the molecular basis of CRN-induced changes to plant cells and nuclei in particular, will not only help understand CRN effector function, but also unveil novel nuclear processes that impact on cell death and immunity. We anticipate that ultimately, the study of nuclear effectors is pivotal to appreciate the nuclear processes that help determine infection outcomes.

## Materials and methods

### Bacterial culture growth, culture filtrate preparation procedures, plant growth conditions, and phenotype scoring

For all experiments, *Agrobacterium tumefaciens* strain AGL1 was used as recipient strain for all constructs. AGL1 strains carrying respective constructs were grown in liquid cultures at 28°C (shaking at 225 rpm) until mid-log phase. Optical Density (OD) was measured (at 600 nm) and cells adjusted to relevant densities using infiltration media (described below). *P. capsici* culture filtrates (CFs) were prepared by inoculating liquid pea broth (PB) with mycelial plugs of strain LT1534. Cultures were incubated at 25°C in the dark without agitation for 5 days. CF was prepared by removing the mycelial mat after which the resulting liquid culture was filter sterilized. PB used as negative controls was prepared simultaneously and sterilized before use in PTI assays. *Nicotiana benthamiana* plants were grown in a greenhouse under 16 h of light and maintained at a temperature of ~25/22°C (day/night). For all experiments, 5-week old plants were used and kept under these conditions during the course of the experiment, unless otherwise stated. The level of cell death observed in plants during experiments was visually scored using a scale of 0–6, with a score of 0 indicating no symptoms, and a score of 6 indicating severe black necrotic lesions. This scale was used as described previously (Stam et al., 2013).

### Preparation of fusion constructs

For construction of a GFP fusion construct containing the CRN N-terminus, corresponding gene fragments were amplified using primers 168080-F_BHI (5′-aaaaaggatccccGTGAAAGTGGACGAAGGCGC-3′) and 168080_R_EcoRI (5′-aaaacgaattctaCGGAACCACCACCAGCACGTG-3′). For cloning of the mature gene coding fragment, primers 168080-F_BHI together with 20_624-R (5′-AAAAAGGCGCGCCTTATTGCAGCATCGCGTAAATTTTCCC-3′) and ASC-I-STREPII-TAG (5′-aaaaagcggccGCTCACTTCTCGAACTGCGGGTGCGACCACCGGCGCGCC-3′) were used. *Bam*HI/*Eco*RI and *Bam*HI/*Asc*I digestions were performed for CRN-N terminal and mature protein constructs before ligation into pre-digested pENTR1a vector. Preparation of CRN C-terminal constructs has been described in Stam et al. (2013). pENTR1A-CRN constructs were sequence verified and used for recombination into the binary vector pB7WGF2 (Karimi et al., 2002), carrying a 35S promoter element and N-terminal GFP-fusion, using Gateway LR reactions (Life Technologies). Constructs were sequence verified before transformation into *A. tumefaciens* strain AGL1.

### CRN induced cell death assays

All EGFP-CRN effector domain fusion and control constructs were generated previously and prepared for infiltration as described in Stam et al. (2013). For cell death assays with CRN20_624 N-terminus, C-terminus and mature fusion proteins, all relevant cultures were adjusted to an OD of 1.0. Cultures were then mixed 1:1 with *A. tumefaciens* AGL1 cells carrying the silencing suppressor P19 at an OD of 1.0, giving a final OD of 0.5 for each CRN and 0.5 for P19. For experiments aimed to compare the kinetics of cell death induction upon ectopic expression of CRN20_624 (DN17), CRN83_152 (DXZ), and CRN79_188 (D2), ODs were adjusted to 0.5 for each culture and mixed with P19 in a 1:1 ratio (giving a final OD of 0.25). This OD proved to be optimal for monitoring cell death simultaneously for all of the CRNs. Plants were infiltrated with the bacterial suspensions and the level of cell death scored up to 7 days post-infiltration (dpi) as described above. Ten to twenty-five individual spot infiltrations were used per construct and all experiments were repeated at least three times. Means for the three CRN constructs were compared for each time point using One-Way ANOVA with SPSS Statistics 21. Graphs show average values for one representative experiment. In a complementary experiment, ion leakage measurements were taken during the time course. For each measurement, 8 leaf disks were harvested from *N. benthamiana* plants infiltrated as described above, and placed together in 10 ml of Milli Q H_2_O and shaken at room temperature at 75 rpm for 2 h. After this time, total dissolved solids (TDS) were measured in the solution using a Primo pocket TDS tester (Hanna Instruments). For each time point and treatment, 6 individual measurements were taken from plants grown in 2 separate greenhouses.

### PTI assays

*A. tumefaciens* AGL1 cells carrying EGFP-CRN fusion constructs were prepared and used for infiltrations as described above using a final OD of 0.25 for each effector. After 48 h, leaf panels were infiltrated with either CF generated from *P. capsici* liquid cultures or a control solution of PB media prepared as described above. Development of symptoms was recorded and the level of cell death was scored 48 h after CF treatment. We infiltrated and scored ten leaves for each construct as described above. The experiment was conducted three times. Statistical analysis was done using SPSS Statistics 21. Equality of the means was tested for each relevant pair of treatments, using the *t*-test with independent samples.

### PTI marker gene expression analyses

For qRT-PCR analyses, leaf panels expressing EGFP prepared as above, were treated with CF or a control solution of PB. After this second infiltration, 3 leaf discs (around 75 mg of tissue) were collected from individual plants at three different time points (1, 3, and 12 h post CF/PB infiltration). Tissues were then used for RNA extraction using the RNeasy plant mini kit (Qiagen). RNA was treated using the DNA-free kit (Ambion) following the manufacturers protocol. cDNA was synthesized using superscript III reverse transcriptase kit (Invitrogen). qPCR was performed using the Power SYBR Green kit (Applied Biosystems) following manufacturer's instructions. The primer pairs used are described in Nguyen et al. (2010) and have previously been used successfully for *P. infestans* CF (McLellan et al., Accepted): NbEF1α-F (5′-TGGACACAGGGACTTCATCA-3′) and NbEF1α-R (5′-CAAGGGTGAAAGCAAGCAAT-3′), NbPti5-F (5′-CCTCCAAGTTTGAGCTCGGATAGT-3′) and NbPti5-R (5′-CCAAGAAATTCTCCATGCACTCTGTC-3′), NbAcre31-F (5′-AATTCGGCCATCGTGATCTTGGTC-3′) and NbAcre31-R (5′-GAGAAACTGGGATTGCCTGAAGGA-3′), and NbGras2-F (5′-TACCTAGCACCAAGCAGATGCAGA-3′) and NbGras2-R (5′-TCATGAGGCGTTACTCGGAGCATT-3′).

The following cycle conditions were used for all primers: initial denaturation at 95°C for 15 min, followed by 40 cycles at 95°C for 15 s and 60°C for 1 min, with a plate read after each cycle. Melt curve reads were performed every 1°C between 60 and 95°C and held for 5 s. Expression levels of each gene induced by CF were calculated relative to expression in leaves mock-infiltrated with PB. Expression of marker genes was normalized to the *N*bEF1α endogenous control gene.

### ETI assays

*A. tumefaciens* AGL1 cells carrying GFP-CRN fusion constructs, empty vector (EV), dexamethasone-inducible Avr3a^KI^ (in pBAV105), R3a and the silencing suppressor P19 were prepared for infiltration as described above. Cultures carrying CRN, EV, and P19 constructs were diluted to a final OD of 0.25, and those harboring Avr3a^KI^ and R3a were adjusted to a final OD of 0.1 before infiltration of plants. An OD of 0.1 was chosen for Avr3a^KI^ and R3a since higher ODs prevented an accurate comparison of the level of cell death between the three CRNs. For conditional expression of Avr3a^KI^, 30 mM dexamethasone (DEX) in 0.1% Tween 20 was infiltrated into leaves 48 h after initial *Agrobacterium* infection as described by Engelhardt et al. (2012). As a negative control, we co-expressed R3a with the allelic variant Avr3a^EM^, which is not recognized by R3a (Bos et al., 2009). Development of Avr3a^KI^-R3a dependent cell death on CRN expressing leaves was assessed 24 h after DEX treatment, scored and tested for significance as described above.

### Western blotting

Plant tissue was harvested 2, 3, and 4 dpi from infiltrated sites and frozen in liquid nitrogen. Protein extractions were performed on ground tissue using GTEN buffer (10% Glycerol, 25 mM Tris, 1 mM EDTA, 150 mM NaCl) supplemented with 2% PVPP, 10 mM DTT, and 1X Complete protease inhibitor cocktail (Thermo Scientific). Samples were run on Biorad TGX gels before transfer to PVDF membranes using the Biorad Trans Blot Turbo Transfer System. Blots were blocked for 30 min with 5% milk in TBS-T (0.1% Tween 20), probed with StrepII-HRP antibody (1:5000) (Genscript) to detect CRNs, and then washed three times in TBS-T for 5 min before incubation with Millipore Luminata Forte substrate. Images were collected on a Syngene GBox TX4 Imager. Blots were then re-probed with GFP antibody (Cambio) followed by anti Mouse-HRP antibodies (Santa Cruz) (1:20000), to detect free EGFP, and washed three times in TBS-T for 5 min before being imaged as before.

### Confocal imaging

For confocal microscopy, *A. tumefaciens* cells were resuspended in infiltration buffer (25 mM MgCl2 and 150 μM acetosyringone) to a final OD of 0.05—0.1 enabling CRN visualization while reducing the risk of observing over-expression artifacts. Control localizations with free EGFP were carried out using plants infiltrated with EV. For nucleolar imaging, *A. tumefaciens* GFP-CRN suspensions were combined 1:1 with *A. tumefaciens* cells carrying a RFP-Fibrillarin expression construct (Goodin et al., 2007) to give a final OD of 0.05 for the CRN and 0.05 for RFP-Fibrillarin. Confocal imaging was carried out 48 h post-infiltration. For DAPI staining, leaves were infiltrated with 4′,6-Diamidino-2-Phenylindole dilactate (Invitrogen) at a final concentration of 5 μg/ml. Subnuclear localization was examined on a Zeiss LSM 710 confocal microscope with a W Plan-Apochromat 40× /1.0 DIC M27 water dipping lens and using the following settings: GFP (488 nm excitation and 495–534 nm emission), mRFP (561 nm excitation and 592–631 nm emission) and DAPI (405 nm excitation and 415–481 nm emission). Cell viability was monitored during CRN localization using transmitted light detection. Confocal imaging for localization of the N- and C-terminus and mature protein was carried using *A. tumefaciens* at an OD of 0.1 and using a Leica SP2 with HCX APO L U-V-I 63.0× water dipping lens with 488 nm excitation wave length.

### OMX 3D-SIM imaging

For OMX imaging, *A. tumefaciens* AGL1 cells transformed with GFP-CRN fusion constructs (CRN20_624, CRN83_152, and CRN79_188) were grown and prepared as described above to a final OD of 0.05. The bacterial suspensions were infiltrated into leaves of 5 week old *N. benthamiana* H2B-RFP transgenic plants (Martin et al., 2009) and *N. tabacum* plants grown and kept in the greenhouse as described above. OMX imaging was carried out 48 h post-infiltration. Epidermal peels were harvested from infiltrated leaf panels and placed immediately into an agarose pad (*N. benthamiana*) or in 70% glycerol (for *N. tabacum*) for imaging. OMX 3D-SIM was performed as described in Posch et al. (2010).

## Results

### CRN20_624 induced cell death and localization only requires the C-terminal effector domain

**Figure 1:**
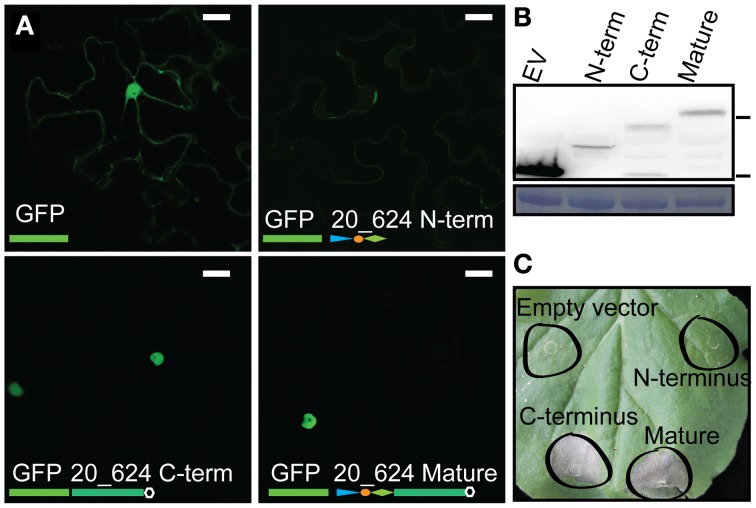
**CRN induced cell death and nuclear localization is conferred by the C-terminus. (A)** Localization of ectopically expressed CRN-GFP fusion products. The panels show the localization for free eGFP, CRN20_624 N-terminus, CRN20_624 C-terminus, and mature CRN20_624 protein, 2 days post infiltration at OD 0.1 Scale bar = 25 μm. **(B)** Immunoblot analysis of CRN20_624 upon over-expression in plant tissue. The blot was probed with anti-GFP antibody. 70 and 25 kDa markers are indicated on the right hand side. Lower panel shows coomassie brilliant blue staining loading control. **(C)** Cell death inducing activity of CRN-GFP fusion products 7 days after infiltration at an OD of 1.0.

CRN effectors are modular proteins harboring a conserved N-terminus required for translocation and C-terminal regions carrying effector activities (Haas et al., 2009; Schornack et al., 2010; Liu et al., 2011; Stam et al., 2013). Given their modularity and a possible impact of CRN N-termini on effector function, we assessed whether the N-terminus of CRN20_624 alters localization or cell death inducing activity. To assess and compare localization of the CRN20_624 N-terminus, the C-terminal effector domain as well as the mature protein were fused to EGFP, expressed in *N. benthamiana* leaves and localized by confocal microscopy in epidermal cells (Figure 1). Both mature protein and the C-terminal domain exclusively localized to the nucleus, suggesting that the CRN C-terminus drives nuclear localization of mature CRN protein (Figure 1A). Consistent with this, expression of the EGFP-tagged N-terminal domain contrasted specific nuclear accumulation as this domain was found distributed throughout the cell, resembling distribution of free EGFP in the cytosol (Figure 1A). We used Western blot analysis to confirm that all EGFP-CRN domain fusions were expressed to similar levels *in planta*. Besides protein levels, these analyses revealed that resultant proteins were largely stable in plant cells as only low levels of EGFP cleavage was observed (Figure 1B). To test whether the presence of the N-terminus affects CRN induced cell death, we infiltrated *N. benthamiana* leaves with all CRN20_624 fusion constructs and the EV control. These experiments showed that both the mature protein and the C-terminus of CRN20_624 induce cell death at similar levels (Figure 1C). Consistent with our localization experiments, expression of the CRN N-terminus and GFP control only resulted in mild chlorosis. These data confirm that the CRN C-terminus is sufficient for nuclear accumulation and cell death inducing activity. Furthermore, these results suggest that the CRN N-terminus does not contribute to or impede effector activity once inside host cells.

### Ectopic expression of CRN effector domains leads to different levels of cell death

**Figure 2:**
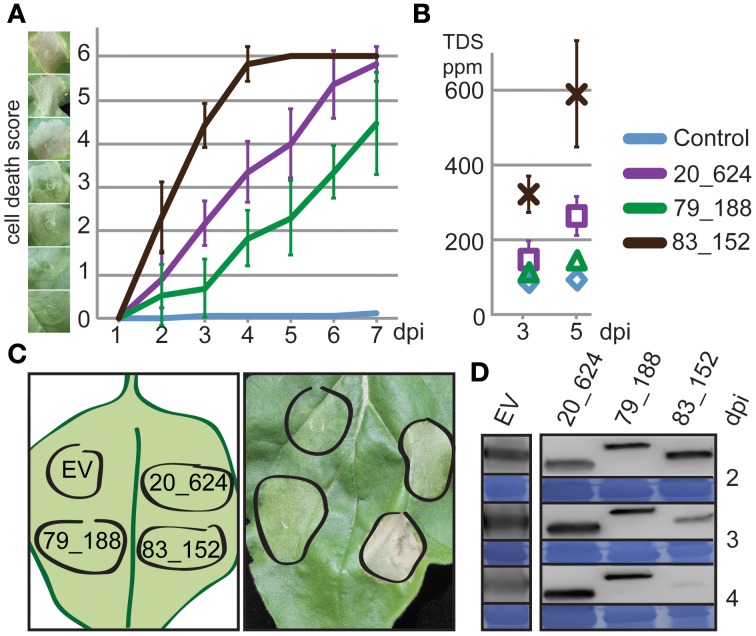
**Necrosis inducing CRNs show distinct cell death inducing dynamics. (A)** Progression of cell death in *N. benthamiana* leaves infiltrated with CRNs. Cell death was scored every 24 h on a scale of 0–6. Example lesions for each score are shown on the left. The graph shows average values ± standard deviation for one representative experiment. **(B)** Ion leakage measurements confirming differences in cell death response. Each data point is an average of 6 measurements ± standard deviation. TDS ppm: total dissolved solids in parts per million. **(C)** Graphical representation of the experimental set-up (left) and a typical leaf 4 days post inoculation. **(D)** Western blots and loading control for CRNs and control samples showing protein levels up to 4 dpi. EV: GFP antibodies, CRNs: strepII antibodies.

Previously, we have shown that CRN20_624, CRN79_188, and CRN83_152 C-termini, classified as DN17, D2, and DXZ domains, respectively, induce cell death upon ectopic over-expression in *N. benthamiana* (Stam et al., 2013). Given that these three CRNs induce cell death but differentially affect *P. capsici* virulence in infection assays (Stam et al., 2013) we elected to compare and contrast CRN induced cell death phenotypes in more detail. To assess whether there are differences in cell death inducing activity, we expressed each CRN effector domain in *N. benthamiana* and scored for cell death across different time points (Figure 2). Assessment of cell death occurring from 1–7 days showed significant differences in the timing and level of cell death between the CRNs from day 2 to day 7 (ANOVA *p* < 0.01) (Figure 2A). Expression of CRN83_152 led to a fast cell death response, reaching maximum levels (6) within 4 days of agro-infiltration, whereas CRN79_188 only induced marginal levels of cell death in the course of this experiment. Compared to CRN83_152 and CRN79_188, CRN20_624 exhibited an intermediate phenotype in these assays. *Post-hoc* Bonferroni tests show that cell death scores for all three CRN proteins were significantly different (*p* < 0.05) on almost all days except for day 2, when CRN20_624 and CRN79_188 show no activity yet and day 7, where CRN20_624 and CRN83_152 both reached maximum cell death scores. We excluded the possibility of variation between leaves by expressing all CRNs and the EV on the same leaf (Figure 2C) and using multiple leaves in multiple experiments. To independently verify the levels of CRN induced cell death, we repeated these experiments and measured levels of ion leakage at 3 and 5 days (Figure 2B). Levels of ion leakage in infiltrated leaves differed significantly between CRN and EV constructs at both days and was consistent with macroscopic evaluation of CRN induced cell death (Figure 2A). CRN83_152 caused the greatest level of ion leakage determined by measuring TDS, while CRN79_188 caused ion leakage at levels just above those for the EV control. CRN20_624 expression led to ion leakage at levels between those seen for CRN83_152 and 79_188. Beyond 5 days it was not possible to measure ion leakage accurately, due to the advanced state of tissue necrosis. Given the possibility that differences in cell death induction are due to levels of CRN proteins, we measured and compared EGFP-CRN levels in a typical experiment at day 2, 3, and 4. Western blots (Figure 2D) showed slight variation in expression levels between CRN constructs, which was not correlated to cell death levels. Given the differences in levels of cell death induction and the similar levels of EGFP-CRN found accumulating in our experiments, we conclude that CRN induced cell death phenotypes are distinct and may reflect different effector activities.

### CRN20_624 expression has an additive effect on pamp but not effector induced cell death

**Figure 3:**
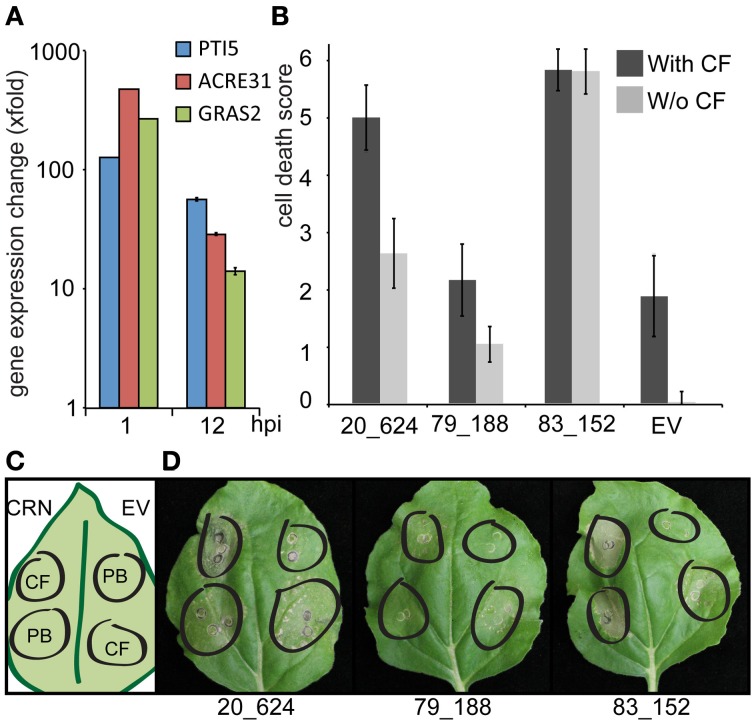
**Expression of CRN20_624, but not CRN79_188 and CRN83_152 has an additive effect on PAMP induced cell death. (A)** qRT-PCR analyses on cDNA derived from leaf panels transiently expressing EGFP and treated with PB and CF. Each bar represents the fold change in gene expression upon CF treatment relative to PB ± standard deviation. Expression was examined for known PTI marker genes *NbPti5*, *NbAcre31*, and *NbGras2* at 1 and 12 h post infiltration (hpi). **(B)** Graph showing average necrosis scores ± standard deviation for three independent experiments. **(C)** Graphical representation of the experimental set-up. CRN-GFP fusion constructs were infiltrated into *N. benthamiana* plants and after 48 h, leaves were infiltrated with either a PAMP cocktail (*Phytophthora capsici* culture filtrate) or a control solution of pea broth. Cell death was scored on a scale of 0–6, 48 h after CF treatment. **(D)** Examples of representative leaves for each treatment on day of scoring.

Given their proposed roles as virulence factors and the distinct differences in CRN sequence, cell death induction and subnuclear localization, we asked whether CRN effector activity leads to perturbation of host PTI or ETI signaling pathways. To test for effects on PAMP induced cell death, *N. benthamiana* leaf panels expressing EGFP-CRN fusion proteins and EGFP were infiltrated with *P. capsici* derived CFs and PB as negative control (Figure 3). Treatment of agro-infiltrated leaf panels with CF leads to PTI induction as qRT-PCR analyses on cDNA derived from EGFP-expressing leaf panels, treated with PB or CF, showed significant induction of PTI marker genes *NbPti5*, *NbAcre31*, and *NbGras2* when compared to expression in PB treated tissues at 1 and 12 h, respectively (Figure 3A). Moreover, leaf panels expressing EGFP showed a specific cell death response to CF since infiltration of PB did not result in visible cell death (Figures 3B,D). Control experiments in which leaf panels expressing the *P. infestans* effector AVR3a were treated with CF, led to reduced cell death, suggesting suppression of CF induced response to PAMPs (data not shown). Interestingly, expression of CRN20_624 was found to have an additive effect on cell death induced by CF treatments in our experiments (Figure 3B). Direct comparisons of cell death between EV and CRN20_624 expressing leaf panels showed a significant increase of cell death (*p* < 0.01), which contrasted results obtained with CRN79_188. Although CRN79_188 induced some cell death without CF treatment, the combination of CRN79_188 with CF did not result in a stronger cell death response when compared to the EV control (*p* = 0.8) (Figure 3B). In these assays, we were not able to assess the effect of CRN83_152 on PTI since we could not find significant differences in the levels of cell death between CF treatment and the PB control (*t*-test for equality of means, *p* = 1) (Figure 3B). These results indicate that the CRN effector activities leading to cell death are distinct and in the case of CRN20_624, intersect with other cell death pathways in plants.

**Figure 4:**
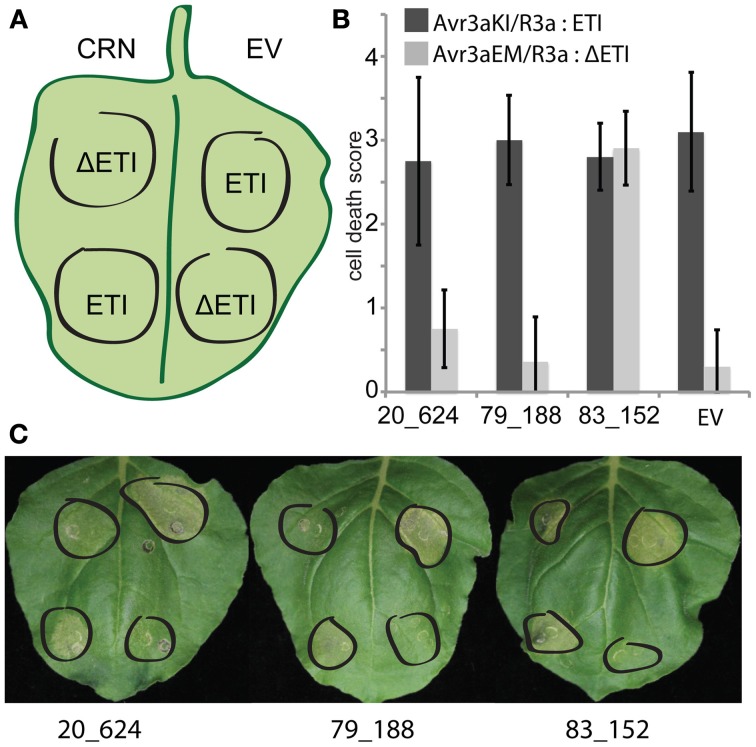
**Necrosis inducing CRNs do not cause altered ETI responses within the plant. (A)** Graphical representation of the experimental set-up. CRN-GFP fusion and EV constructs were co-infiltrated into *N. benthamiana* leaves with Avr3a^KI^ and R3a (ETI), and Avr3a^EM^ and R3a (ΔETI) to monitor ETI responses. After 48 h, leaves were infiltrated with dexamethasone and incubated for a further 24 h for induction of Avr3a expression. **(B)** Cell death in response to CRNs in ETI and non-ETI induced leaves scored on a scale of 0–6. Graph shows average necrosis scores ± standard deviation for one representative experiment. **(C)** Examples of representative leaves for each treatment on day of scoring.

Given that CRN20_624 has an additive effect on cell death upon CF treatment, we asked whether any of our effectors affect ETI mediated cell death (Figure 4). To test this, we over-expressed CRN20_624, CRN83_152, and CRN79_188 in *N. benthamiana* leaves with R3a whilst also introducing *P. infestans* Avr3a^KI^ and Avr3a^EM^ coding genes under a DEX inducible promoter. In these assays, Avr3a^EM^ served as a negative control, as it is not recognized by R3a (Bos et al., 2009). Co-infiltration of CRN fusion proteins and EGFP in combination with R3a and AVR3a constructs, allowed us to express CRN fusion proteins with R3a first before activating Avr3a^KI^ induced ETI with DEX treatment. Phenotypic assessment of leaf panels 24 h after DEX induction revealed robust HR development. In these assays, there was no evidence of either enhanced or reduced ETI responses in CRN expressing leaves based on direct comparisons to our EV controls (ANOVA, *p* = 1). These results suggest that the presence of these CRN effectors does not affect ETI induced cell death. As expected, induction of AVR3a^EM^ expression in the presence of R3a did not lead to HR demonstrating that the observed cell death was due to specific recognition of AVR3a^KI^. From these results, we conclude that CRN20_624 specifically promotes PAMP induced cell death. We suggest that the contrasting observations between CRN proteins reflect differences in effector functions, each of which leads to cell death upon ectopic expression.

### CRN effector domains feature distinct subnuclear localization and their ectopic expression causes distinct changes in host nuclear morphology

**Figure 5:**
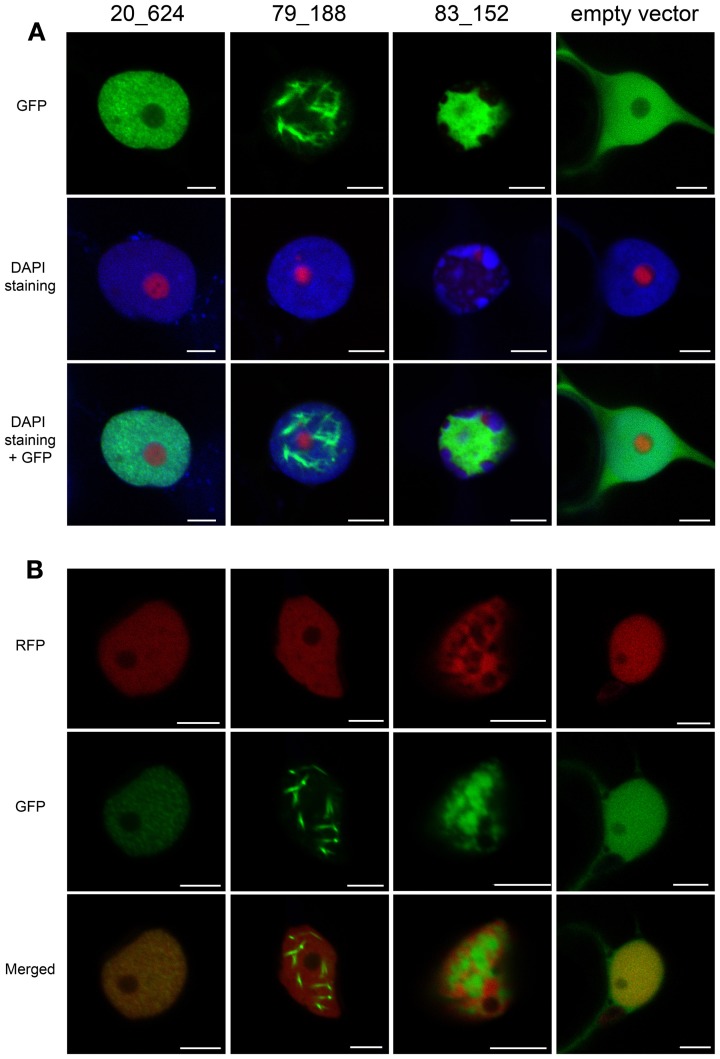
**CRN 83_152 causes re-localization of DNA within the nucleus**. CRN-GFP fusion constructs were over expressed in *N. benthamiana* plants and imaged by confocal microscopy 48 h post-infiltration. **(A)** Leaves were co-infiltrated with RFP-fibrillarin and were DAPI stained by infiltrating with 4′,6-Diamidino-2-Phenylindole dilactate at a final concentration of 5 μg/ml. **(B)** *N. benthamiana* plants stably expressing histone RFP were infiltrated with CRN-GFP fusion constructs as described above. Scale bars = 5 μm.

We have presented evidence suggesting that CRN83_152, CRN20_624, and CRN79_188 feature distinct cell death phenotypes and differentially affect cell death pathways. Given their distinct localization patterns upon over-expression (Stam et al., 2013) we asked whether localization of nuclear markers during CRN expression would allow further insights into the onset of cell death in plants. Confocal microscopy was used to determine the nuclear localization of EGFP-CRN proteins as well as the nucleolar marker Fibrillarin and nuclear DNA. CRN20_624 showed a clustered distribution pattern confined to the nucleoplasm that contrasted localization of CRN79_188. Expression of CRN79_188 consistently led to the detection of filament-like structures in the nucleus. In contrast, CRN83_152 was present in patches within the nucleus, with clear areas of nuclear space in which EGFP-CRN83_152 protein appeared absent (Figure 5). These patterns were observed in living cells as cytoplasmic streaming was evident in cells expressing all EGFP-CRN fusions (Supplementary videos 1–4). Interestingly, distribution of DAPI stained nuclear DNA appeared altered in cells expressing CRN83_152 (Figure 5A), suggesting re-localization of host chromatin.

To confirm this observation, we expressed EGFP-CRN83_152 in transgenic *N. benthamiana* plants carrying histone-RFP (Figure 5B). These experiments revealed that consistent with our observation on DAPI stained DNA, over-expression of CRN83_152 caused Histone 2B-RFP labeled DNA to accumulate in distinct patches within the nucleus. In these assays, CRN83_152 was found to accumulate in areas in the nucleus from which DNA had been excluded. Consequently, CRN83_152 and DAPI/Histone 2B-RFP signal did not co-localize in both of our experiments (Figures 5A,B). In contrast to CRN83_152, over-expression of CRN20_624 and CRN79_188 did not alter the distribution of DNA. DAPI and histone-RFP signal were detected evenly within the nuclear space, with only some small patches where DNA was absent, similar to the pattern observed for cells expressing free GFP (Figures 5A,B). To exclude the possibility of changes in nuclear morphology after cell death, we repeated these assays whilst confirming cell viability by assessing cytoplasmic streaming and vesicle movement within the cytoplasm during CRN and EGFP expression. In these experiments, nuclear re-organization caused by expression of CRN83_152 did not appear to affect cell viability within the time scale of these experiments (Supplementary video 3).

### 3D-SIM imaging of CRN effectors reveals distinct localization within the nucleus

**Figure 6:**
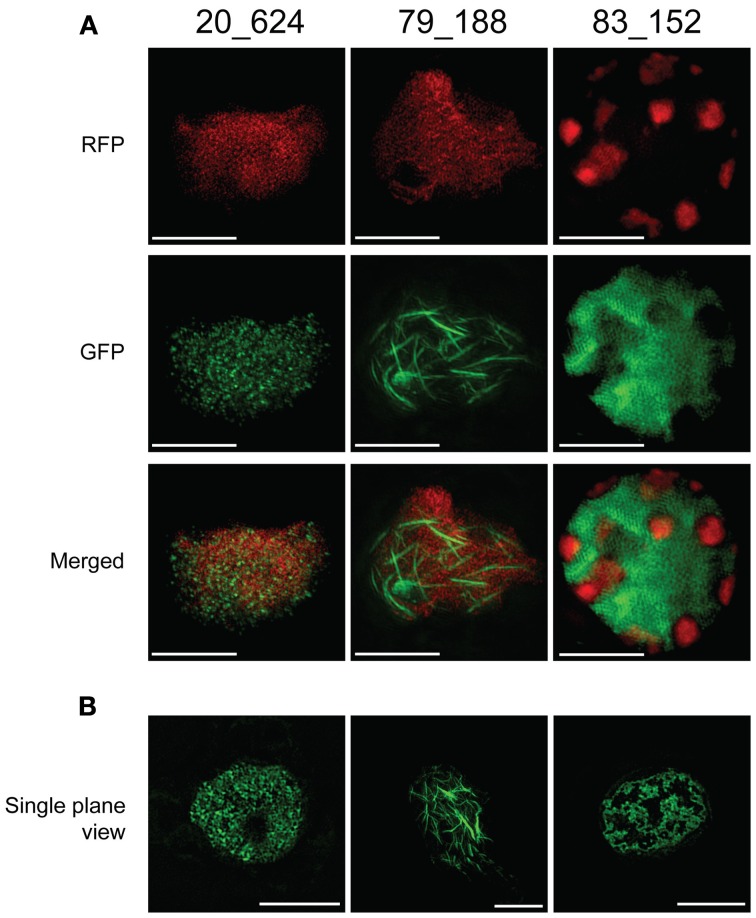
**3D-SIM imagining of CRN C-terminal constructs on OMX confirms distinct localization within the nucleus. (A)** Projection view of 3D-SIM images on epidermal peels of *N. benthamiana* H2B-RFP transgenic lines infiltrated with CRN-GFP fusion constructs. Histone 2B distribution is shown in RFP channel whereas CRN protein distribution is in the GFP channel. Merged channel confirms impairment of chromatin distribution in CRN83_152 expressing cells. **(B)** Single plane views of 3D-SIM images on epidermal peels of *N. tabacum* infiltrated with CRN-GFP fusion constructs show distinct nuclear distribution patterns for CRN effector proteins. All images were taken 48 h after infiltration. Scale bars = 5 μm.

Using confocal microscopy, we observed distinct subnuclear localization and structures upon expression of the three CRN effectors characterized in this study (Figure 5). To gain a better understanding of these results, we used super-resolution 3D structured illumination microscopy (3D-SIM) to visualize the possible structures CRN proteins form or interact with at the subnuclear level (Figure 6). 3D-SIM imaging of *N. benthamiana* leaves expressing Histone 2B-RFP and EGFP-CRN fusions confirmed localization patterns observed in our confocal microscopy experiments for CRN83_152 and CRN79_188 (Figure 5). CRN20_624 was found distributed in clusters throughout the nucleoplasm in close proximity to Histone 2B-RFP labeled chromatin (Figure 6A). CRN79_188 was found to form regular and evenly distributed fibril-like structures interspersed with chromatin (Figure 6B). In both cases, distribution of chromatin in the nucleus is not impaired. High-resolution images, however, shines a different light on CRN83_152 localization. Whereas confocal images suggest that CRN83_152 localizes in a uniform manner in patches within the nucleoplasm, OMX microscopy reveals that these patches consist of long and undulating strands, surrounding areas of re-localized chromatin. This is particularly evident in single plane images (Figure 6B) and could not be observed with confocal microscopy.

Although the mechanism of chromatin exclusion or degradation in the presence of CRN83_152 is yet elusive, our results suggest that one mechanism of cell death induction could rely on the modification of chromatin affecting its integrity and consequently, disrupting important host cell processes.

## Discussion

CRN effectors are considered a diverse and ubiquitous class of effectors found in all plant pathogenic oomycetes sequenced to date. Consequently, various studies have hinted at a role in virulence, suppression of PTI and more recently, ETI (Liu et al., 2011; Van Damme et al., 2012; Shen et al., 2013; Stam et al., 2013). Here we provide further evidence of functional diversity amongst *P. capsici* CRN proteins by studying the activity of three necrosis-inducing effectors. Consistent with previous studies, we show that for CRN20_624, the N-terminal region does not affect cell death induction or localization, suggesting that only the C-terminal effector domain is required for function in the nucleus. Although only shown for CRN20_624, this work further supports the observation that nuclear localization is required for cell death induction as shown for *P. infestans* CRN8 (Schornack et al., 2010) and that CRN C-termini carry the cell death inducing activity (Torto et al., 2003; Haas et al., 2009). We demonstrate that based on timing and intensity of cell death as well as their effects on CF triggered cell death, CRN20_624, CRN79_188, and CRN83_152 have distinct activities *in planta*.

Macroscopic evaluation of cell death as well as ion leakage measurements upon CRN expression revealed that CRN83_152 expression causes rapid cell death and tissue collapse, whereas CRN79_188 causes delayed cell death and CRN20_624 features an intermediate phenotype. Western blot analyses revealed that all EGFP-CRN fusions accumulated to similar levels, suggesting that differences in cell death reflect distinct activities rather than effector abundance. This observation is further illustrated by the diverse CRN localization patterns as well as distinct changes in nuclear morphology and DNA distribution upon CRN83_152 accumulation.

Besides cell death induction, we have presented evidence that CRN20_624, but not CRN83_152 and CRN79_188, has an additive effect on PAMP induced cell death. Treatment of CRN expressing leaves with either PB or CFs showed a marked increase in cell death on CF treated panels, suggesting modification of PAMP induced cell death signaling. These results contrasted with cell death induced by recognition of the *P. infestans* effector AVR3a by R3a. Importantly, these results could suggest that CRN20_624 activity induces specific cell death pathways, excluding those associated with ETI. If true, this would mean that CRN proteins could be used to classify and study PAMP triggered nuclear signaling pathways. CRN20_624 mediated promotion of PTI is counter-intuitive as effectors are generally thought of as suppressors of PTI. It is possible, however, that PTI stimulation represents a virulence function in the late stages of a hemi-biotrophic lifecycle, when cell death and tissue collapse is apparent (Jupe et al., 2013). Interestingly, CRN20_624, which contains the DN17 C-terminal domain, is expressed at later stages during infection, coinciding with the switch of *P. capsici* from a biotrophic to a necrotrophic lifestyle (Stam et al., 2013). This adds additional weight to a model in which *P. capsici* deploys effectors to co-opt host PTI signaling pathways and promote cell death. If true, the identification and engineering of CRN20_624 host targets may allow reduction of cell death during *P. capsici* infection and slow disease progression.

Consistent with diverse functions, we reveal distinct subnuclear localization patterns for the CRN effectors studied here. Detailed co-localization studies of CRN83_152, CRN20_624, and CRN79_188 together with DAPI staining as well as nucleolar and chromatin markers, not only confirmed the organization of EGFP-CRN proteins in distinct patterns, but unveiled unexpected changes in the organization of nuclear chromatin upon expression of CRN83_152. Multiple localization experiments showed that CRN83_152 occupies the nuclear space around DAPI and H2B-RFP labeled patches of DNA. 3D-SIM high resolution microscopy not only confirmed these observations but added additional detail, showing organization of CRN83_152 in intricately organized convoluted structures, wrapping around or in close proximity to nuclear chromatin. At this stage, we do not know the molecular basis or function of CRN83_152. Although we have previously shown that CRN83_152 enhances *P. capsici* virulence, we do not know the relevance of chromatin re-organization toward immunity or susceptibility. Studies currently on the way in our group will aim to identify the principal targets for CRN83_152 and study their role in immunity. It is likely that these studies will help unveil novel processes underpinning *Phytophthora* virulence.

In contrast to CRN83_152, microscopy revealed that CRN79_188 is distributed in long thin filamentous strands. Importantly, we found that these filaments are uniform and evenly distributed throughout the nucleoplasm. These results suggest that CRN79_188 either forms these structures by itself or interacts with yet unknown structures in the nucleus. In this regard, the recent identification of F-actin containing structures in plant cells containing the Turnip Vein Clearing Virus movement protein MP-TVCV (Levy et al., 2013), raises this possibility.

Based on our results, we question as to whether cell death induction is a direct virulence function or rather, is a feature that is an indirect consequence of (distinct) effector activities. Ectopic expression in plant cells led to rapid accumulation of CRN proteins in *N. benthamiana* cells to levels that are unlikely to occur *in vivo* during infection. We also cannot exclude that perception of bacterial PAMPs has an impact on our results in the case of our cell death assays. However, leaf panels expressing EGFP remained healthy, showing low levels of ion leakage and were responsive to CF treatment as evidenced by induction of PTI marker genes and occurrence of CF-specific cell death in our experiments. Whether priming of defence responses affect levels of cell death or not, the differences in cell death kinetics for the CRN effectors tested were consistent and significant across our experiments. Because of the necessity for both an epitope tag and fluorescent reporter, we have used EGFP-CRN protein fusions for this work. We can therefore not formally exclude the possibility that the presence of N-terminal EGFP affects CRN function or activity levels. Given the observations that CRN proteins are modular in nature and mature CRN proteins also feature sizeable N-terminal region that does not appear to affect function or localization for CRN20_624 (Figure 1), this is not a likely scenario.

Taken together, our work suggests distinct differences in cell death mediated by diverse CRN effector activities. These findings are thus consistent and build on previous work, which showed differential effects of CRN over-expression on *P. capsici* virulence (Stam et al., 2013). This study further supports the emerging view that through yet unknown mechanisms this ancient class of effector proteins act on processes required for plant immunity. With an increasing number of nuclear host defence signaling components identified in plants together with pathogen effectors that target the nucleus, there is a critical need to understand the nuclear processes that drive plant immunity and ETS. Our results strongly suggest that exploring the functions of CRN effectors, including those that induce cell death, will uncover immunity-associated nuclear processes in the host. Given the enormous diversity of nuclear effectors now identified in the oomycetes, these proteins form a rich source of molecular probes suited to study nuclear biology. CRN effectors and other nuclear effectors will thus emerge as valuable tools to unravel nuclear processes involved in plant immunity.

## Conflict of interest statement

The authors declare that the research was conducted in the absence of any commercial or financial relationships that could be construed as a potential conflict of interest.

## Supplementary material

Supplementary video 1 **Time-lapse video of cells expressing CRN 20_624**. *N. benthamiana* plants were infiltrated with CRN 20_624 at a final OD of 0.05 and video images captured 48 h post-infiltration using transmitted light detection. Videos show vesicle movement within the cytoplasm during CRN expression. Video was taken using a 40× water dipping lens and a 3.2× zoom (Scale bar = 20 μm).

Supplementary video 2 **Time-lapse video of cells expressing CRN 79_188**. *N. benthamiana* plants were infiltrated with CRN 79_188 at a final OD of 0.05 and video images captured 48 h post-infiltration using transmitted light detection. Videos show vesicle movement within the cytoplasm during CRN expression. Video was taken using a 40× water dipping lens and a 3.3× zoom (Scale bar = 20 μm).

Supplementary video 3 **Time-lapse video of cells expressing CRN 83_152**. *N. benthamiana* plants were infiltrated with CRN 83_152 at a final OD of 0.05 and video images captured 48 hours post-infiltration using transmitted light detection. Videos show vesicle movement within the cytoplasm during CRN expression. Video was taken using a 40× water dipping lens and a 1.2× zoom (Scale bar = 20 μm).

Supplementary video 4 **Time-lapse video of cells expressing pB7WGF2 empty vector (EV)**. *N. benthamiana* plants were infiltrated with EV at a final OD of 0.05 and video images captured 48 h post-infiltration using transmitted light detection. Videos show vesicle movement within the cytoplasm during free GFP expression. Video was taken using a 40× water dipping lens and a 4.6× zoom (Scale bar = 20 μm).
